# Gender inequalities in violence victimization and depression in Brazil: results from the 2019 national health survey

**DOI:** 10.1186/s12939-023-01916-4

**Published:** 2023-05-24

**Authors:** Matías Mrejen, Leonardo Rosa, Dayana Rosa, Thomas Hone

**Affiliations:** 1 Instituto de Estudos para Políticas de Saúde (IEPS), São Paulo, Brazil; 2 Instituto de Medicina Social, Universidade do Estado de Rio de Janeiro (IMS UERJ), Rio de Janeiro, Brazil; 3 Department of Primary care and Public Health, Imperial College London, South Kensington, UK

**Keywords:** Gender inequalities, Depression, Violence victimization, Brazil

## Abstract

**Background:**

Violence is a worldwide public health challenge and has been linked to depression in many settings. Depression is higher in women and differential exposure to violence is a potential risk factor – especially in countries with high-levels of violence. This paper provides a comprehensive characterization of the association between violence victimization and depression in Brazil, focusing on sex/gender inequalities.

**Methods:**

We used data from the 2019 wave of the National Health Survey (PNS) in Brazil to assess whether respondents had depression (using PHQ-9) and if they were victims of violence, differentiating by the type of violence, the frequency of victimization, and the primary aggressor. We used logit models to assess the association between victimization and the likelihood of having depression. We predicted probabilities of being depressed, considering the interaction between violence victimization and sex/gender, to analyze the differences between men and women.

**Results:**

Rates of violence victimization and depression were higher among women than among men. The odds of being depressed were 3.8 (95%CI: 3.5–4.2) times higher among victims of violence than among non-victims, and 2.3 (95%CI: 2.1–2.6) times higher among women than among men, adjusting for socioeconomic factors. For any given income level, racial/ethnic or age group, victims of violence who were women had the highest predicted probabilities of being depressed – e.g., 29.4% (95%CI: 26.1–32.8) for lower-income women, 28.9% (95%CI: 24.4–33.2) for black women, and 30.4% (95%CI: 25.4–35.4) for younger women that suffered violence. Over one in three women that suffered multiple types of violence, experienced violence more frequently, or where the aggressor was an intimate partner or another family member were predicted to have depression.

**Conclusions:**

Being a victim of violence was strongly associated with higher risk of depression in Brazil, with women more likely to be both victims of violence and develop depression. Frequent, sexual, physical or psychological violence, and intimate partners or family member perpetrators were major risk factors for depression and should be a public health priority.

**Supplementary Information:**

The online version contains supplementary material available at 10.1186/s12939-023-01916-4.

## Background

Violence is a worldwide public health concern. Reducing lethal and non-lethal violence is target 16.1 and eliminating all violence against women and girls is target 5.2 of the United Nations’ Sustainable Development Goals (SDGs) [1, 2]. Lethal violence accounts for 1.3% of Years of Life Lost worldwide, [3, 4] and non-lethal violence can also have severe consequences [5–8]. For instance, non-lethal violence increases the risk of mental disorders (anxiety, depression, and others) [5, 9, 10]. Non-lethal violence affects specific demographic groups more, such as women, younger individuals, and people experiencing racial discrimination (such as Afrodescendants and ethnic minorities) [11, 12]. In particular, women are more likely to suffer intimate partner violence, which is also associated with poorer mental health [12, 13].

The association between being the victim of non-lethal violence and a higher probability of depression is well established by previous research, including evidence from Brazil [13–18]. Evidence suggests that this association is possibly bidirectional. On the one hand, individuals with mental health disorders are at higher risk of being victims of violence – they may be targeted due to their vulnerabilities or they may put themselves in riskier situations [19–21]. On the other hand, exposure to violence is a traumatic event which can lead to stress, fear, and isolation, factors that may lead to depression [20, 21].

The prevalence of depression is higher among women than men, and evidence suggests that differential exposure to violence might be a contributing factor [13, 16, 19]. However, comprehensive characterizations of gender inequalities in violence victimization and depression in low- and middle-income countries are lacking – especially those with high levels of violence such as Brazil. This is particularly relevant as the higher prevalence of depression and other common mental disorders among women can be attributed to the interaction of different adversities, like poverty, gender disempowerment, gender-based violence, and sexual harassment [22, 23].

Brazil is a relevant context for studying gender inequalities in violence victimization and depression. Exposure to violence is highly prevalent and a major public health concern. In 2019, the prevalence of violence victimization among adults was 18.3%, and was higher among women, black and brown/mixed race individuals, and younger adults (aged 18 to 29) [24]. That same year, the prevalence of depression among the adult population was 10.8% with women 7.1% points more likely to be depressed than men [15]. Additionally, evidence suggests that violence victimization contributes to higher rates of depression among women than among men [16]. However, gender inequalities in the types of violence experienced, by perpetrator and severity, and their links with depression are not well understood in Brazil and a better understanding is vital to inform health policy.

This paper provides a comprehensive characterization of the relationship between being a victim of violence and depression in Brazil, and explores gender inequalities, using data from a nationally representative health survey. We estimate gender inequalities in the prevalence of depression and violence victimization, overall and according to the violence suffered (i.e., type, frequency, and aggressor). We analyze the relationship between violence victimization and depression by gender using probabilities predicted from results of regression models to adjust for socioeconomic and demographic factors.

## Materials and methods

### Data

We used data from the 2019 wave of the National Health Survey (PNS). The PNS is a cross-sectional and nationally representative household-based survey, administered by the Brazilian Institute of Geography and Statistics (IBGE) in partnership with the Ministry of Health. Data collection obeyed a three stages sampling design. First, it randomized census tracts within each state. Second, it randomized the participants’ households within the selected census tracts. Finally, in each household, it randomized one individual (15 years old or older) to answer an in-depth questionnaire on health, including self-perception of health status, lifestyle, and violence victimization. Microdata made available by the IBGE contains all information needed to account for the sampling design, including weights adjusted for non-response rates and population projections [25].

We used two main variable groups from the detailed questionnaire: the Patient Health Questionnaire and the questionnaire on violence victimization. The Patient Health Questionnaire (PHQ-9) is a standard instrument extensively used for screening depression, [26] previously validated for Brazilian respondents [27]. The PHQ-9 questionnaire asks the individual how often over the last two weeks they have been bothered by symptoms of depression: “not at all” (score: 0), “less than half the days” (score: 1), “more than half the days” (score: 2), or “nearly every day” (score: 3). More specifically, questions about the following symptoms are included: “little interest or pleasure in doing things”; “feeling down, depressed, or hopeless”; “trouble falling or staying asleep, or sleeping too much”; “feeling tired or having little energy”; “poor appetite or overeating”; “feeling bad about yourself—or that you are a failure or have let yourself or your family down”; “trouble concentrating on your usual activities”; “moving or speaking slowly; or being fidgety and restless”; “thoughts that you would be better off dead, or thoughts of hurting yourself in some way”. The total score for each individual is computed by summing the score for each symptom and indicates the severity of depression (0–4 none, 5–9 mild, 10–14 moderate, 15–19 moderately severe, 20–27 severe) [26–28].

The questions on violence victimization combine a series of Yes/No questions regarding exposure to violence in the previous 12 months. This includes questions about five forms of physical violence, five forms of psychological violence, and two forms of sexual violence. Table A1 in the Supplementary Material shows the list of questions included in each group. For each group of questions, individuals reported who the primary aggressor was, and the frequency that it happened (once / sometimes / many times).

Additionally, we used data on family income per capita, sex/gender, race/ethnicity, age, highest educational level achieved, area of residence (urban/rural), state of residence, economic activity status, employment status, number of residents in the household, if the partner lives in the same household, registration with a Family Health Team (FHT, Brazil’s main public primary health care provider), frequency of home visits received from any member of a FHT in the last 12 months, private health insurance, and consumption of tobacco products, physical activity in the last three months, and frequency of alcohol consumption.

We excluded individuals younger than 18, as the PHQ-9 is validated for the adult population [26]. We also excluded nine observations with missing data on race/ethnicity, and 22 with missing data on household income. After exclusion, 88,500 individuals were included in our sample.

### Variables

We computed variables identifying depression and victimization, as well as a list of covariates, for all individuals.

#### Dependent variable: depression

For depression, we computed the PHQ-9 score and classified individuals with a total score ≥ 10 as depressed. This cut-off is frequently used as a sign of clinically relevant symptoms of depression – e.g., a study in Brazil found the PHQ-9 had a sensitivity of 72.5% and a specificity of 88.9% for diagnosing depression using this cut-off [27]. We also computed the share of depressed individuals that were depressed but not treated.

#### Independent variable: violence victimization

For victimization, we used the violence questionnaire to create a variable identifying individuals that have suffered at least one violent episode in the previous 12 months. We also generated three binary variables identifying victimization by the type of violence suffered, i.e., physical, psychological, and sexual.

To assess the association between depression and victimization according to the type of violence, we created a categorical variable indicating if the individual had suffered only physical/sexual/psychological violence, two types of violence, or the three types of violence. To assess the association between depression and victimization according to the primary aggressor, we created a categorical variable indicating if in at least one violent episode the primary aggressor was the current or former intimate partner (“Partner”), if in no episode the primary aggressor was the current or former intimate partner and in at least one was another family member (“Family and not partner”), if in none of the violent episodes suffered the aggressor was a current or former intimate partner or another family member (“Other”) or if the person had not suffered any violent episode in the last 12 months (“None”). To analyze the association between the frequency of victimization and depression, we divided individuals into four groups, by frequency of victimization: high, moderate, low, and none. In our classification, high-frequency individuals reported having suffered one type of violence many times or reported having suffered more than one type of violence sometimes. Moderate-frequency individuals are those that suffered one type of violence sometimes or suffered more than one type of violence once. Low-frequency victims are those that suffered only one type of violence once in the last 12 months. Non-victims are those that have not suffered any type of violence.

#### Covariates

We included the following individual characteristics to explore how the association between violence and depression varies across individuals: sex/gender (male/female), income quintile (according to family income per capita), race/ethnicity (white, brown/mixed, black, indigenous, Asian), and age group (18–24, 25–34, 35–44, 45–54, 55–64, 65 or more). Finally, we added covariates that can be related to the prevalence of depression and/or victimization: region of residence (North, North-East, Center-West, South-East, South), education (Basic incomplete, Basic complete, Secondary complete, Higher complete), marital status (single, married, divorced, widow), cohabitation (lives alone, with an intimate partner, with another person), physical activity (no, less than weekly, once or twice a week, three or more times a week), alcohol consumption (no, once a week, more than once a week), a series of binary variables indicating if the person is employed / lives in an urban area / is registered in a public primary healthcare facility / is covered by private healthcare insurance / smokes tobacco.

### Statistical analyses

We computed prevalence estimates (using survey weights) and 95% confidence intervals for all variables included in our analyses according to sex/gender (women/men) and victimization (women that suffered violence, women that did not suffer violence, men that suffered violence, women that did not suffer violence). To assess inequality, we also computed the prevalence of depression and of victimization for men and women separately by income quintile, race/ethnicity, and age.

To assess the relationship between victimization and depression, we estimated logistic models with depression as a dependent variable and included different forms of victimization and the set of covariates described above as independent variables. With post-regression prediction, we computed the predicted probabilities of being depressed, considering the interaction between violence victimization and sex/gender, to analyze the differences between men and women. Also, we separate estimates by different forms of victimization and/or relevant socioeconomic characteristics from models that included interaction terms between all variables of interest. We report odds ratios from analogous models without those interactions among predictors (to keep the results easily interpretable) in the Supplementary Material.

All analyses were performed using Stata (version 16.1) employing survey weights.

## Results

Table 1 presents summary statistics for women (53% of total observations) and men (47%). Compared to men, women in the sample were older, of lower income, more likely to be divorced or widowed, and not living with a partner, and of higher. Women had higher rates of both violence victimization and depression than men. While 19.4% of women (95%CI: 18.7–20) suffered at least one violent episode in the last 12 months and 15% (95%CI: 14.4–15.6) presented symptoms compatible with depression; those shares were 17% (95%CI: 16.3–17.7) and 6.1% (95%CI: 5.7–6.5) among men, respectively (Table 1).

**Table 1:** Summary statistics

	Women						Men					
	All		Suffered violence		Did not suffer violence		All		Suffered violence		Did not suffer violence	
	Prop.	95%CI	Prop.	95%CI	Prop.	95%CI	Prop.	95%CI	Prop.	95%CI	Prop.	95%CI
Violence	0.194	0.187–0.200					0.170	0.163–0.177				
Depression	0.150	0.144–0.156	0.312	0.292–0.332	0.111	0.105–0.117	0.061	0.057–0.065	0.144	0.128–0.161	0.044	0.040–0.047
*Type of violence*												
Psychological violence	0.186	0.179–0.192	0.959	0.952–0.966			0.160	0.153–0.167	0.940	0.930–0.949		
Physical violence	0.042	0.038–0.046	0.219	0.200–0.237			0.040	0.037–0.044	0.238	0.220–0.256		
Sexual violence	0.010	0.008–0.013	0.054	0.043–0.065			0.004	0.003–0.006	0.026	0.019–0.032		
Only physical	0.006	0.005–0.007	0.030	0.024–0.036			0.009	0.008–0.011	0.056	0.047–0.065		
Only sexual	0.002	0.001–0.003	0.010	0.007–0.014			0.001	0.001–0.001	0.004	0.003–0.006		
Only psychological	0.145	0.139–0.151	0.748	0.729–0.768			0.127	0.121–0.134	0.748	0.729–0.766		
Two types	0.037	0.033–0.041	0.191	0.173–0.210			0.031	0.028–0.034	0.181	0.164–0.197		
All types	0.004	0.003–0.005	0.020	0.015–0.025			0.002	0.001–0.003	0.011	0.007–0.016		
*Primary aggressor*												
Family (not partner)	0.052	0.049–0.056	0.270	0.254–0.287			0.028	0.025–0.031	0.165	0.150–0.181		
Intimate partner violence	0.064	0.059–0.068	0.329	0.309–0.349			0.026	0.023–0.029	0.152	0.136–0.169		
Other	0.078	0.073–0.082	0.401	0.382–0.420			0.116	0.110–0.122	0.683	0.663–0.702		
*Frequency of violence*												
Low	0.066	0.062–0.070	0.339	0.320–0.357			0.071	0.065–0.076	0.415	0.391–0.438		
Moderate	0.087	0.082–0.092	0.448	0.429–0.467			0.074	0.069–0.079	0.436	0.414–0.459		
High	0.041	0.038–0.045	0.213	0.198–0.228			0.025	0.023–0.028	0.149	0.135–0.163		
*Age*												
18–24	0.130	0.130–0.130	0.205	0.190–0.220	0.112	0.108–0.116	0.149	0.149–0.149	0.243	0.224–0.261	0.129	0.125–0.133
25–34	0.175	0.170–0.179	0.204	0.191–0.217	0.168	0.162–0.173	0.188	0.184–0.192	0.231	0.213–0.248	0.180	0.174–0.185
35–44	0.203	0.197–0.209	0.228	0.212–0.243	0.197	0.191–0.204	0.201	0.195–0.208	0.213	0.195–0.230	0.199	0.192–0.206
45–54	0.180	0.175–0.184	0.167	0.153–0.180	0.183	0.177–0.188	0.177	0.172–0.183	0.146	0.131–0.160	0.184	0.177–0.190
55–64	0.153	0.148–0.157	0.116	0.106–0.127	0.161	0.155–0.167	0.148	0.143–0.153	0.106	0.094–0.118	0.157	0.151–0.162
65 or older	0.160	0.157–0.163	0.081	0.072–0.090	0.179	0.175–0.183	0.136	0.133–0.139	0.062	0.055–0.070	0.152	0.148–0.155
*Income quintile*												
1st	0.212	0.206–0.219	0.266	0.249–0.283	0.200	0.193–0.207	0.187	0.181–0.193	0.204	0.186–0.222	0.184	0.177–0.190
2nd	0.206	0.199–0.212	0.219	0.204–0.234	0.202	0.195–0.210	0.201	0.193–0.208	0.201	0.184–0.218	0.201	0.192–0.209
3rd	0.200	0.193–0.206	0.171	0.158–0.185	0.206	0.199–0.214	0.193	0.186–0.199	0.188	0.170–0.207	0.193	0.186–0.201
4th	0.202	0.195–0.209	0.181	0.163–0.199	0.207	0.199–0.215	0.221	0.213–0.228	0.220	0.200–0.239	0.221	0.213–0.229
5th	0.180	0.172–0.188	0.163	0.148–0.178	0.185	0.176–0.193	0.199	0.190–0.208	0.188	0.170–0.205	0.201	0.192–0.211
*Race/ethnicity*												
Asian	0.009	0.007–0.011	0.009	0.004–0.014	0.009	0.007–0.011	0.010	0.008–0.011	0.006	0.002–0.010	0.010	0.008–0.012
Black	0.114	0.109–0.119	0.125	0.114–0.136	0.111	0.105–0.117	0.116	0.110–0.121	0.135	0.120–0.149	0.112	0.106–0.117
Brown/Mixed	0.433	0.425–0.441	0.452	0.433–0.471	0.429	0.420–0.438	0.443	0.434–0.452	0.477	0.453–0.501	0.437	0.428–0.446
Indigenous	0.005	0.004–0.006	0.007	0.004–0.010	0.004	0.003–0.005	0.006	0.005–0.008	0.007	0.004–0.009	0.006	0.004–0.008
White	0.439	0.431–0.448	0.407	0.388–0.426	0.447	0.438–0.456	0.425	0.416–0.435	0.376	0.354–0.398	0.435	0.426–0.445
*Region of residence*												
Center-West	0.075	0.075–0.075	0.073	0.067–0.079	0.075	0.074–0.077	0.077	0.077–0.077	0.075	0.068–0.082	0.077	0.075–0.078
North	0.076	0.076–0.076	0.077	0.070–0.083	0.076	0.075–0.078	0.081	0.081–0.081	0.080	0.072–0.087	0.081	0.080–0.083
North-East	0.266	0.266–0.266	0.275	0.262–0.288	0.264	0.261–0.267	0.262	0.262–0.262	0.266	0.251–0.281	0.262	0.259–0.265
South	0.145	0.145–0.145	0.134	0.124–0.144	0.148	0.146–0.151	0.149	0.149–0.149	0.134	0.122–0.146	0.152	0.149–0.154
South-East	0.437	0.437–0.437	0.441	0.424–0.459	0.436	0.432–0.440	0.431	0.431–0.431	0.445	0.423–0.468	0.429	0.424–0.433
*Marital status*												
Married	0.397	0.390–0.404	0.321	0.303–0.339	0.415	0.406–0.424	0.486	0.478–0.495	0.374	0.354–0.394	0.510	0.501–0.518
Divorced	0.086	0.082–0.090	0.106	0.095–0.116	0.081	0.077–0.085	0.054	0.050–0.057	0.062	0.052–0.072	0.052	0.048–0.056
Widow	0.108	0.104–0.112	0.058	0.050–0.066	0.120	0.116–0.125	0.023	0.021–0.026	0.018	0.012–0.023	0.025	0.022–0.027
Single	0.409	0.403–0.415	0.515	0.496–0.534	0.384	0.376–0.391	0.436	0.428–0.444	0.546	0.526–0.567	0.414	0.405–0.422
*Cohabitation (lives with:)*												
Alone	0.078	0.075–0.081	0.066	0.061–0.072	0.081	0.077–0.084	0.071	0.068–0.075	0.085	0.077–0.092	0.069	0.065–0.072
Partner	0.554	0.546–0.561	0.505	0.486–0.524	0.565	0.557–0.574	0.682	0.675–0.690	0.589	0.567–0.611	0.701	0.693–0.709
Other person	0.368	0.361–0.376	0.429	0.410–0.448	0.354	0.346–0.362	0.246	0.239–0.253	0.326	0.303–0.348	0.230	0.222–0.238
*Education (highest level completed)*												
Basic incomplete	0.341	0.333–0.349	0.297	0.279–0.314	0.352	0.343–0.360	0.355	0.347–0.363	0.286	0.267–0.305	0.369	0.361–0.378
Basic	0.134	0.128–0.140	0.151	0.138–0.164	0.130	0.124–0.136	0.157	0.151–0.163	0.181	0.165–0.198	0.152	0.145–0.159
Secondary	0.352	0.345–0.360	0.381	0.362–0.401	0.345	0.337–0.354	0.346	0.337–0.355	0.381	0.358–0.404	0.339	0.330–0.348
Higher	0.173	0.165–0.180	0.171	0.155–0.187	0.173	0.166–0.181	0.142	0.134–0.150	0.152	0.132–0.172	0.140	0.132–0.148
*Physical activity*												
No	0.619	0.611–0.628	0.603	0.585–0.622	0.623	0.614–0.632	0.535	0.525–0.544	0.466	0.444–0.489	0.549	0.539–0.559
Less than weekly	0.012	0.010–0.014	0.020	0.013–0.026	0.010	0.008–0.011	0.020	0.017–0.022	0.026	0.020–0.032	0.019	0.016–0.021
Once or twice a week	0.109	0.103–0.114	0.120	0.107–0.133	0.106	0.101–0.111	0.190	0.183–0.197	0.225	0.206–0.245	0.183	0.175–0.191
Three or more times a week	0.261	0.253–0.268	0.257	0.240–0.273	0.262	0.254–0.270	0.255	0.247–0.264	0.283	0.262–0.303	0.250	0.240–0.259
*Alcohol consumption*												
Never	0.682	0.674–0.690	0.604	0.584–0.624	0.701	0.692–0.709	0.460	0.452–0.469	0.405	0.382–0.429	0.472	0.463–0.481
Less than weekly	0.148	0.143–0.154	0.177	0.163–0.191	0.141	0.135–0.147	0.169	0.162–0.175	0.166	0.150–0.181	0.169	0.162–0.176
Once a week	0.095	0.089–0.100	0.120	0.107–0.133	0.089	0.083–0.094	0.144	0.138–0.150	0.148	0.134–0.163	0.144	0.137–0.150
More than once a week	0.075	0.071–0.079	0.099	0.088–0.109	0.069	0.065–0.074	0.227	0.219–0.234	0.280	0.259–0.302	0.215	0.207–0.223
Employed	0.512	0.504–0.520	0.586	0.569–0.603	0.494	0.485–0.503	0.727	0.720–0.733	0.774	0.756–0.793	0.717	0.709–0.724
Urban area	0.879	0.875–0.883	0.899	0.889–0.908	0.874	0.869–0.878	0.843	0.837–0.848	0.884	0.873–0.896	0.834	0.828–0.840
Smokes tobacco	0.096	0.092–0.101	0.121	0.110–0.131	0.091	0.086–0.096	0.159	0.153–0.166	0.208	0.190–0.226	0.149	0.142–0.156
Private health insurance	0.304	0.295–0.313	0.286	0.267–0.305	0.308	0.299–0.318	0.288	0.278–0.298	0.282	0.259–0.304	0.289	0.278–0.300
Registered in public primary healthcare	0.619	0.607–0.631	0.621	0.599–0.642	0.619	0.606–0.632	0.611	0.598–0.623	0.586	0.561–0.610	0.616	0.603–0.629

Note: the table shows the proportion (mean) and 95% confidence intervals of all the variables of interest and covariates used in the analyses. Proportions are presented according to sex/gender and to victimization. All data were weighted according to the PNS survey design and survey weights

The victimization for both psychological violence – 18.6% (95%CI: 17.9–19.2) vs. 16% (95%CI: 15.3–16.7) – and sexual violence – 1% (95%CI: 0.8–1.3) vs. 0.4 (95%: 0.3–0.6) – were also higher among women than among men (the difference in the prevalence of sexual lifetime victimization was larger, as shown in Figure A1 in the Supplementary Material). However, the rates of experiencing physical violence were not statistically different between men and women. Women and men victims of violence also differed in their aggressors. For women, current or former intimate partners and other family members were the primary aggressors in over half of cases, but they represented less than one-third of cases for cases of violence among men. By the frequency of violence, women were victimized more often than men – over one fifth of victimized women suffered violence with a high frequency (Figure A2 in the Supplementary Material). For most income, race/ethnic or age groups, both violence victimization and depression were higher among women. Prevalence of both indicators are particularly high among specific demographic groups of women: lower income, black and indigenous (Figure A3 in the Supplementary Material).

We present fully adjusted models in Table A2 in the Supplementary Materials, showing that sex/gender, income, age, and race/ethnicity are systematically associated with a higher likelihood of being the victim of a violent episode. Compared to men, women were 1.3 (95% CI: 1.2–1.4) times more likely to have suffered any type of violence, 1.4 (95% CI: 1.3–1.5) times more likely to have suffered psychological violence, 1.3 times more likely to have suffered physical violence (95% CI: 1.1–1.5), and 3.2 times more likely (95%CI: 2.3–4.4) to have suffered sexual violence in the last 12 months. Table A3 in the Supplementary Material show that the odds of being depressed were 3.8 (95%CI: 3.5–4.2) times higher among victims of violence than among non-victims, and 2.3 (95%CI: 2.1–2.6) higher among women than among men.

**Fig. 1:**
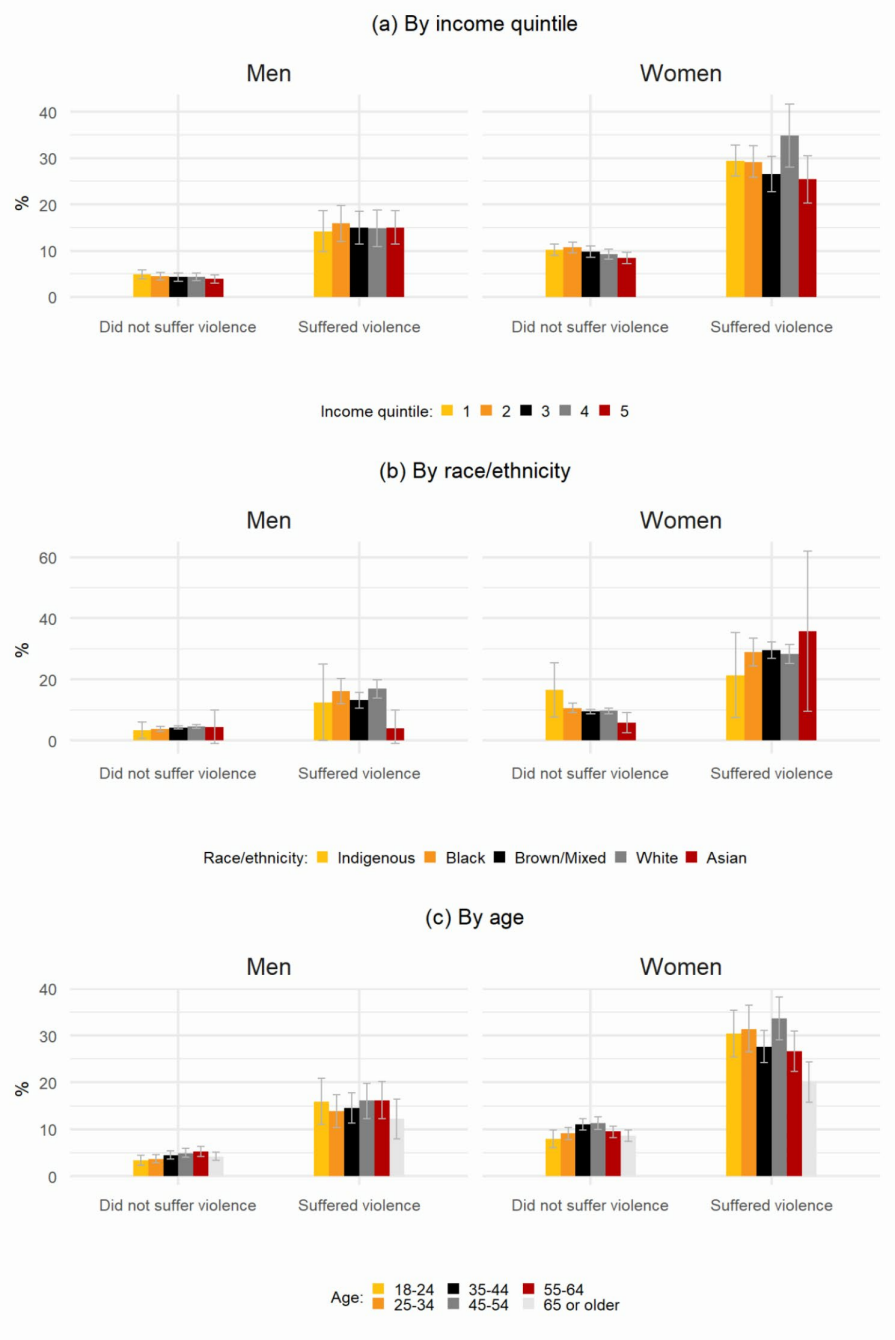
Predicted probabilities of being depressed by sex/gender and violence victimization, according to sociodemographic characteristics Note: The figure shows the predicted probabilities for being depressed according to sex/gender, victimization and income quintile (panel a), race/ethnicity (panel b), and age (panel c). Victimization was defined as being the victim of at least one violent episode in the past 12 months. Depression was defined as having a PHQ-9 score > = 10. Predicted probabilities were estimated using the results of a *logit* model that included as covariates: sex/gender, victimization, income quintile, race/ethnicity, age, region of residence, area of residence (urban/rural), marital status, cohabitation, employment, education, alcohol consumption, tobacco consumption, physical activity, health insurance and registered in primary healthcare services. In each case, predicted probabilities were estimated from a model that included an interaction term between violence, sex/gender and a sociodemographic characteristic (income, race/ethnicity or age, respectively). All other covariates were fixed at means. Results from an analogous model, without the interaction terms, are depicted in column 1 of Table A3 as odd ratios. All data were weighted according to the PNS survey design and survey weights

From adjusted logistic model estimates, predicted probabilities were generated (Fig. 1). In all interactions/subgroup analyses by income, race/ethnicity and age, violence victimization was associated with between two- and four-times higher probabilities for depression. In all cases, women victims of violence had consistently the highest rates of depression. For example, 10.2% (95%CI: 9–11.4) of the poorest women (income quintile Q1) who did not suffer violence were predicted to have depression compared to 29.4% (95%CI: 26.1–32.8) of the women of the same income quintile that did suffer violence. For men, those predicted probabilities were 4.9% (95%CI: 3.9–5.9) and 14.2% (95%CI: 9.8–18.6), respectively. Among women who identified as black, 10.7% (95%CI: 9.2–12.2) were predicted to have depression if they did not suffer violence and 28.9% (95%CI: 24.4–33.2) were predicted to if they did. For black men, the rates were 3.9% (95%CI: 3–4.7) and 16.1% (95%CI: 12–20.2) respectively. Notably, there were no statistically significant differences in the probabilities of being depressed according to income, race/ethnicity or age among women victims of violence.

We assessed if the association between violence and depression varied by type of violence, who the primary aggressor was, and the frequency. We present fully adjusted models in Table A3 in the Supplementary Materials, showing that individuals that suffered all three types of violence (OR: 8.3, 95%CI: 5.3–13), were aggressed by an intimate partner (OR: 4.4, 95%CI: 3.7–5.2) or other family member (OR: 4.5, 95%CI: 3.8–5.2), and were aggressed more frequently (OR: 6.1, 95%CI: 5.2–7.2) were the most likely to be depressed.

**Fig. 2:**
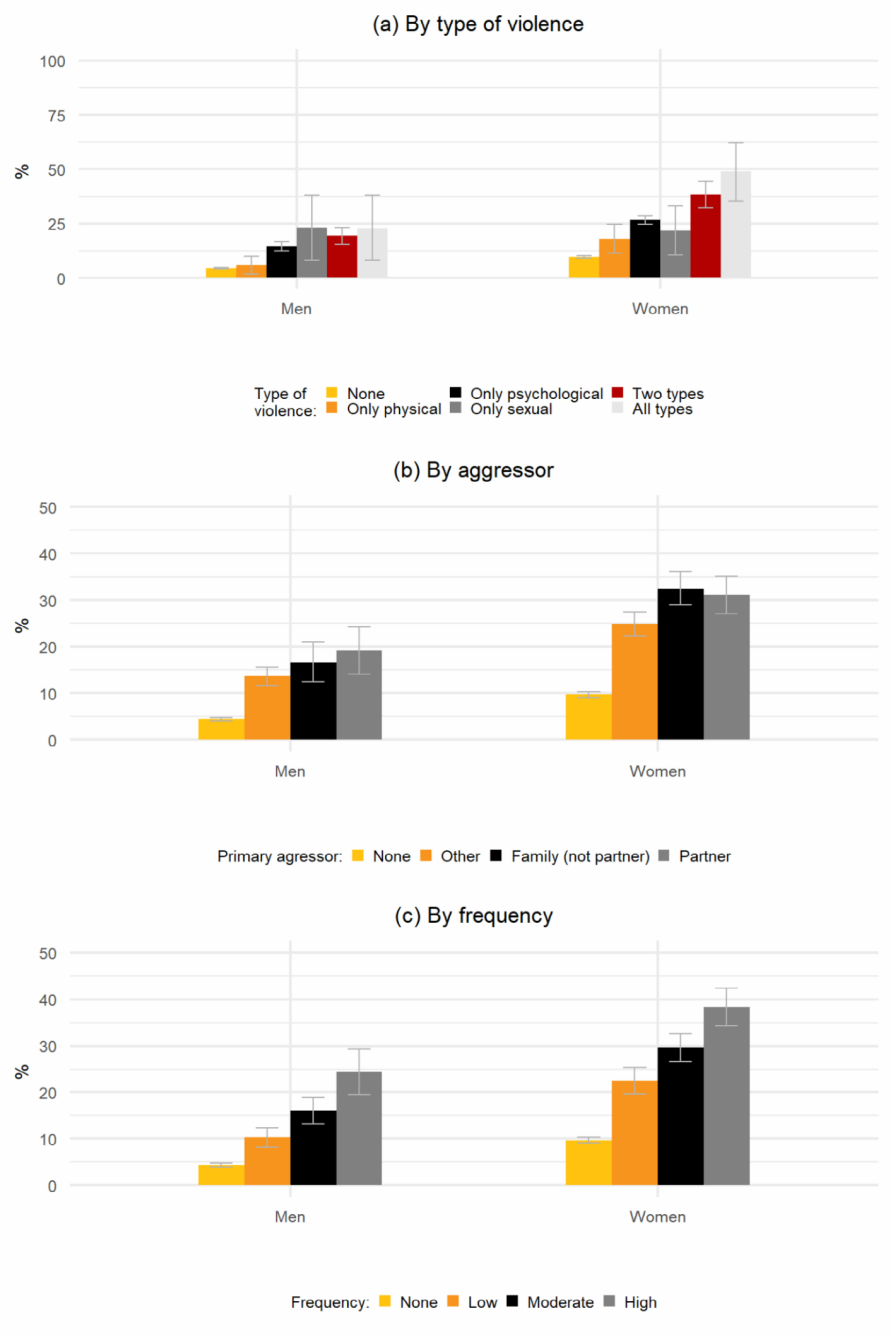
Predicted probabilities for being depressed by sex/gender and violence victimization, according to type of violence, primary aggressor, and frequency of victimization Note: The figure shows the predicted probabilities for being depressed according to sex/gender, and: type of violence suffered (panel a), the primary aggressor (panel b), and frequency of aggressions (panel c). Victimization was defined as being the victim of at least one violent episode in the past 12 months. Depression was defined as having a PHQ-9 score > = 10. Predicted probabilities were estimated using the results of *logit* models that included as covariates: sex/gender, victimization (by type of violence, primary aggressor, and frequency, respectively), income quintile, race/ethnicity, age, region of residence, area of residence (urban/rural), marital status, cohabitation, employment, education, alcohol consumption, tobacco consumption, physical activity, health insurance and registered in primary healthcare services. In each case, predicted probabilities were estimated from a model that included interaction terms between the type of violence and sex/gender (panel a), between the aggressor and sex/gender (panel b), and between frequency and sex/gender (panel c). All other covariates were fixed at means. Results from analogous models, without the interaction terms, are depicted in columns 2 to 4 of Table A3 as odd ratios. All data were weighted according to the PNS survey design and survey weights

Results in Fig. 2 show the predicted probabilities from similar regression models, including interaction terms between the characteristics of violence and sex/gender. Individuals experiencing more than one type of violence, especially women, had higher predicted rates of depression (Panel A, Fig. 2). For example, women suffering sexual, physical and psychological violence, were five times more likely to have depression than women that did not suffer any violence – 48.9% (95%CI: 35.3–62.4) versus 9.7% (95%CI: 9.1–10.3), and three times more likely than victims of physical violence only – 17.9% (95%CI: 11.2–24.7). Women were more likely than men to have depression when suffering physical or psychological violence or when experiencing multiple types of violence.

When exploring the role of the primary aggressor (Panel B, Fig. 2), the predicted depression rates for women who experienced violence by intimate partners or other family members were 31.1% (95%: 27.1–35.1) and 32.5% (95%: 28.9–36.1) respectively, compared to 24.9% (95%: 22.3–27.5) if the aggressor was another person. By the frequency of violence (Panel C, Fig. 2) the predicted were significantly larger for those that had suffered violence with high frequency than for those that had suffered it with low frequency: 38.3% (95%CI: 34.3–42.4) versus 22.5 (95%CI 19.6–25.4), respectively, among women, and 24.4% (95%CI: 18.5–27) versus 10.3% (95%CI: 8.1–12.4), respectively, among men. Notably, the highest predicted rates of depression across all analyses were for women that suffered more types of violence, with higher frequency or were where the aggressor was an intimate partner of family member.

As violence victimization is systematically associated with higher probabilities of being depressed, it is also relevant to assess if it affects the probabilities of falling in the treatment gap – i.e., being depressed but not treated. Results shown in Figure A4 in the Supplementary Material suggest that was not the case. We could not find any statistically significant difference in the probabilities of being depressed and untreated neither between women that did and did not suffer violence nor between men that did and did not suffer violence. If anything, point estimates suggest that the probabilities of being untreated were lower among those that suffered violence, especially women.

## Discussion

Being a victim of violence is highly prevalent in Brazil, especially for women. Nearly 1 in 5 women were victims of violence in that last 12 months. Women were also substantially more like to be victims of psychological and sexual violence compared to men. Women, and also lower-income individuals, were more likely to be victims of violence and have depression.

Individuals who were victims of violence were 3.8 times more likely to have depression than non-victims after adjusting for socioeconomic factors, and the association was significantly larger for individuals that were the victims of many types of violences or victimized more frequently. These results are in line with previous evidence from Brazil showing a strong association between violence victimization and depression [15–18, 29]. A recent study, using the same survey data as ours, found similar patterns in the association according to type of violence suffered, [18] with the differences with our study in magnitudes of the association stemming from different definitions of psychological violence. While theirs included solely the threat of physical violence, ours included all survey questions relating to a broad definition of psychological violence – i.e., all acts that might disturb the emotional health of the respondent, like denigration, ridicule, threats and other forms of nonphysical hostile treatment [30].

In all socioeconomic stratifications, women that suffered violence had consistently higher predicted probabilities of being depressed. Over one in three women that suffered multiple types of violence, experienced violence more frequently, or where the aggressor was an intimate partner or another family member were predicted to have depression.

There are multiple mechanisms that can explain the association between violence victimization and depression. On the one hand, individuals with mental health disorders are at higher risk of being victims of violence – they may be targeted due to their vulnerabilities or they may put themselves in riskier situations [19–21]. On the other hand, exposure to violence is a traumatic event which can lead to stress, fear, and isolation, factors that may lead to depression [20, 21]. Most episodes of major depression are preceded by adverse life events, frequently in proximity with the onset of the episode, and stress produced by such events can lead to the onset of neurobiological mechanisms associated with mental disorders [19, 31, 32]. Additionally, the experience of psychological, sexual and/or physical abuse during childhood and adolescence is an stablished risk factor for developing depression in adulthood [30].

The gender gap in depression rates is a well stablished fact in the literature, stemming from the overlapping of biological, cultural and societal factors [19]. While little evidence exists for risks factors to be specifically linked to depression by sex/gender, different levels of exposure to adverse events explain a substantial parts of the gender gap as women are cumulatively more exposed to stressors and different forms adversity (e.g., gender-based discrimination, lower income, intimate partner violence, and abuse during childhood) throughout the life course [19, 30, 33, 34]. Our findings corroborate this, as we found no evidence of gender-specific susceptibility in the association between victimization and depression (i.e., the association between victimization and depression was similar for women and for men) but we did find overall higher rates of depression and of exposure to violence among women. Importantly, characteristics of episodes of violence associated with a higher likelihood of depression, like suffering violence more frequently or being aggressed by an intimate partner or other family member, were more prevalent among women.

The fact that suffering multiple types of violence and being a victim more frequently, as well as being aggressed by an intimate partner or another relative, were associated with higher probabilities of depression highlights the need to treat violence victimization as a multidimensional issue in further research, rather than focusing solely on binary measures of victimization [13, 14, 35]. The fact that the association between violence victimization and depression is not heterogeneous across sociodemographic characteristics (age, race/ethnicity, and income) highlights the overarching nature of the challenge that violence poses to population mental health. However, it is important to note that the prevalence of both indicators is particularly high among lower income, black and indigenous women, which points to the intersection of gender with other disadvantages based on social status and race/ethnicity [20].

Key strengths of this study are the use of a large, recent, and nationally representative survey, the use of the internationally-validated PHQ-9 screening tool for depression which is independent of medical diagnosis, and of an instrument to measure violence victimization comprehensively (by type, primary aggressor, and frequency). An additional strength of this study is the characterization of inequality patterns in victimization and depression considering the interaction between gender and income quintile, race/ethnicity, and age. However, there are limitations. First, as mentioned above, the study design does not allow us to make causal claims about the relationship between violence victimization and depression. Second, while the PHQ-9 is a widely used instrument and has been shown to have high sensitivity and specificity in Brazil, it does not provide a clinical diagnosis of depression. Third, the PNS only samples individuals in permanent households, and individuals arguably more exposed to violence, such as homeless or incarcerated individuals, are excluded. Fourth, violence victimization might be underestimated, as there is likely to be under-reporting by victims and information on some relevant forms of violence (e.g., obstetric violence) could not be included.

Despite those limitations, this study highlights the relevance of violence victimization as a public health concern: it is highly prevalent, disproportionately affects some populations (e.g., women, younger, black, brown/mixed, and lower-income individuals), and it is strongly associated with depression. This is especially relevant for women’s mental health, as the prevalence of depression is significantly higher among them. In particular, the fact that all types of violence, including psychological violence, are associated with depression is particularly challenging. Identifying the frequency and aggressors in each case is crucial, as those dimensions are relevant for understanding the interplay between violence victimization and mental health. Mental health policies should aim at reducing the prevalence of all types of violence victimization, especially among women, and recognize the urgent need to identify victims of all types of violence and provide support for them.

## Conclusion

Being a victim of violence was associated with an increased risk of depression in Brazil, with women more likely to be both victims of violence and develop depression. Socioeconomic disadvantage and severity of the violence further interacted to exacerbate the risk of depression, particularly for women reenforcing the multidimensional issue of violence and depression. Mental health policies should aim to reduce all types of violence, especially against women, and focus on protecting victims of violence from worsening mental health outcomes.

## Electronic supplementary material

Below is the link to the electronic supplementary material.

Supplementary Material 1

## Data Availability

All data used are publicly available in: https://www.ibge.gov.br/estatisticas/sociais/saude/9160-pesquisa-nacional-de-saude.html?=&t=downloads.

## List of Abbreviations

SDGs: United Nations’ Sustainable Development Goals.
IBGE: *Instituto Brasileiro de Geografia e Estatística* (Brazilian Institute of Geography and Statistics).
PNS: *Pesquisa Nacional de Saúde* (National Health Survey).
PHQ-9: Patient Health Questionnaire.
