# Supplementary material for: Gender inequalities in violence victimization and depression in Brazil: results from the 2019 national health survey
Source: Int J Equity Health. 2023 May 24;22:100. doi: 10.1186/s12939-023-01916-4 (PMC10207796; doi:10.1186/s12939-023-01916-4)
Supplement: Supplementary file 1 — Supplementary Material 1 [file 12939_2023_1916_MOESM1_ESM.docx]

# Supplementary Material

### Table A1 - Questions from the 2019 National Health Survey used for measuring violence victimization

| **Type of violence** | **Questions** |
| --- | --- |
| Psychological | In the last 12 months, did someone humiliate, offend or ridicule you in front of other people? |
|  | In the last 12 months, did someone scream at you or insult you? |
|  | In the last 12 months, did someone use social networks or a cell phone to threaten, offend, insult or expose images without your consent? |
|  | In the last 12 months, did someone threaten to hurt someone important to you? |
|  | In the last 12 months, did someone destroy something yours on purpose? |
| Physical | In the last 12 months, did someone slap you? |
|  | In the last 12 months, did someone push, shove or throw something at you? |
|  | In the last 12 months, did someone punch you, hit you, or kick you? |
|  | In the last 12 months, did someone choke or burn you on purpose? |
|  | In the last 12 months, did someone threaten or hurt you with a weapon? |
| Sexual | In the last 12 months, did someone touch you, kiss you or expose part of your body without your consent? |
|  | In the last 12 months, did someone threaten or force you to have sexual relationships without your consent? |

### Figure A1 - Lifetime victimization (sexual violence)


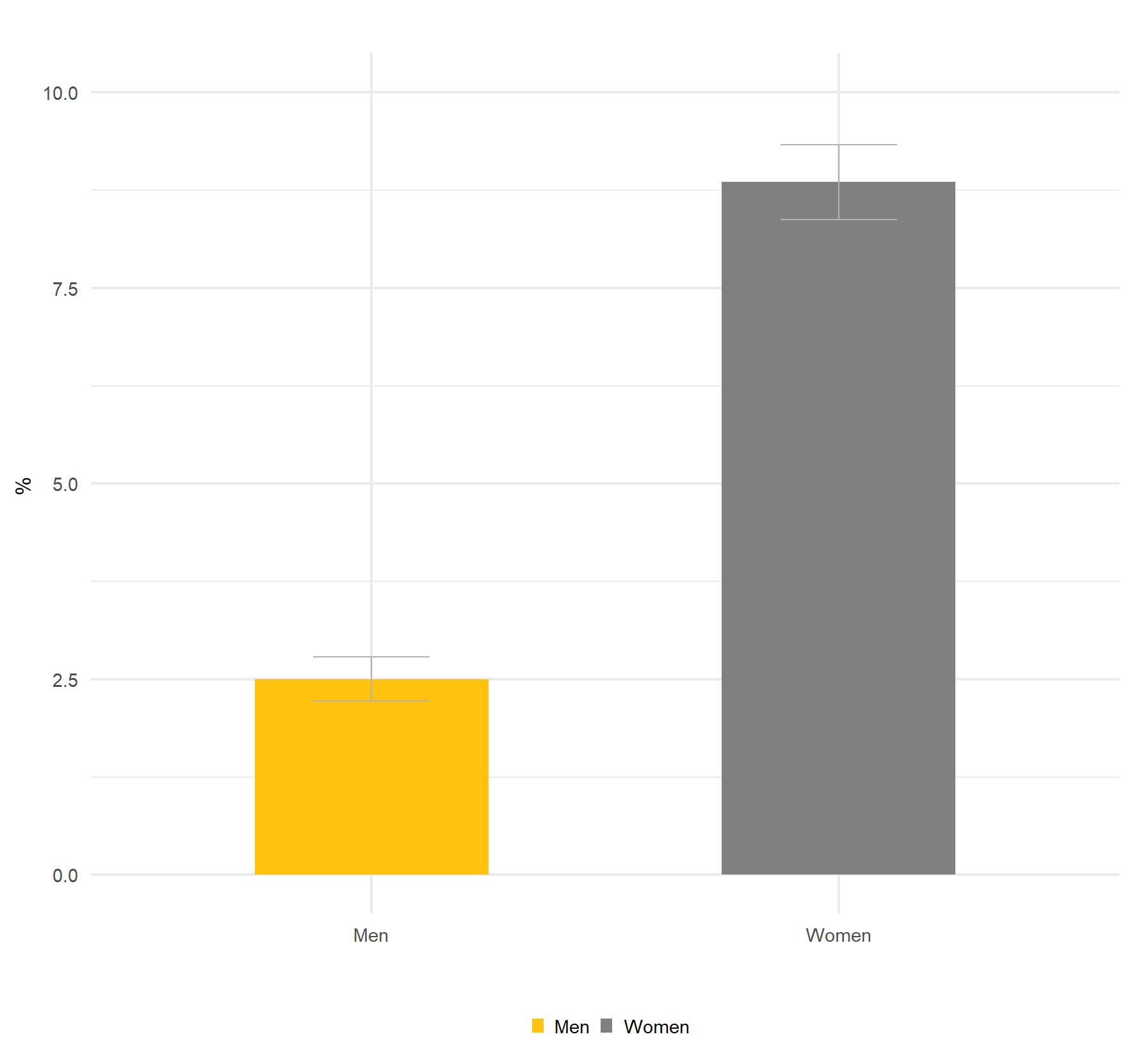


Note: The figure shows the prevalence of lifetime victimization to sexual violence. Victimization was defined as being the victim of at least one episode of sexual violence ever. All data were weighted according to the PNS survey design and survey weights.

### Figure A2 - Frequency of aggressions and primary aggressor by type of violence


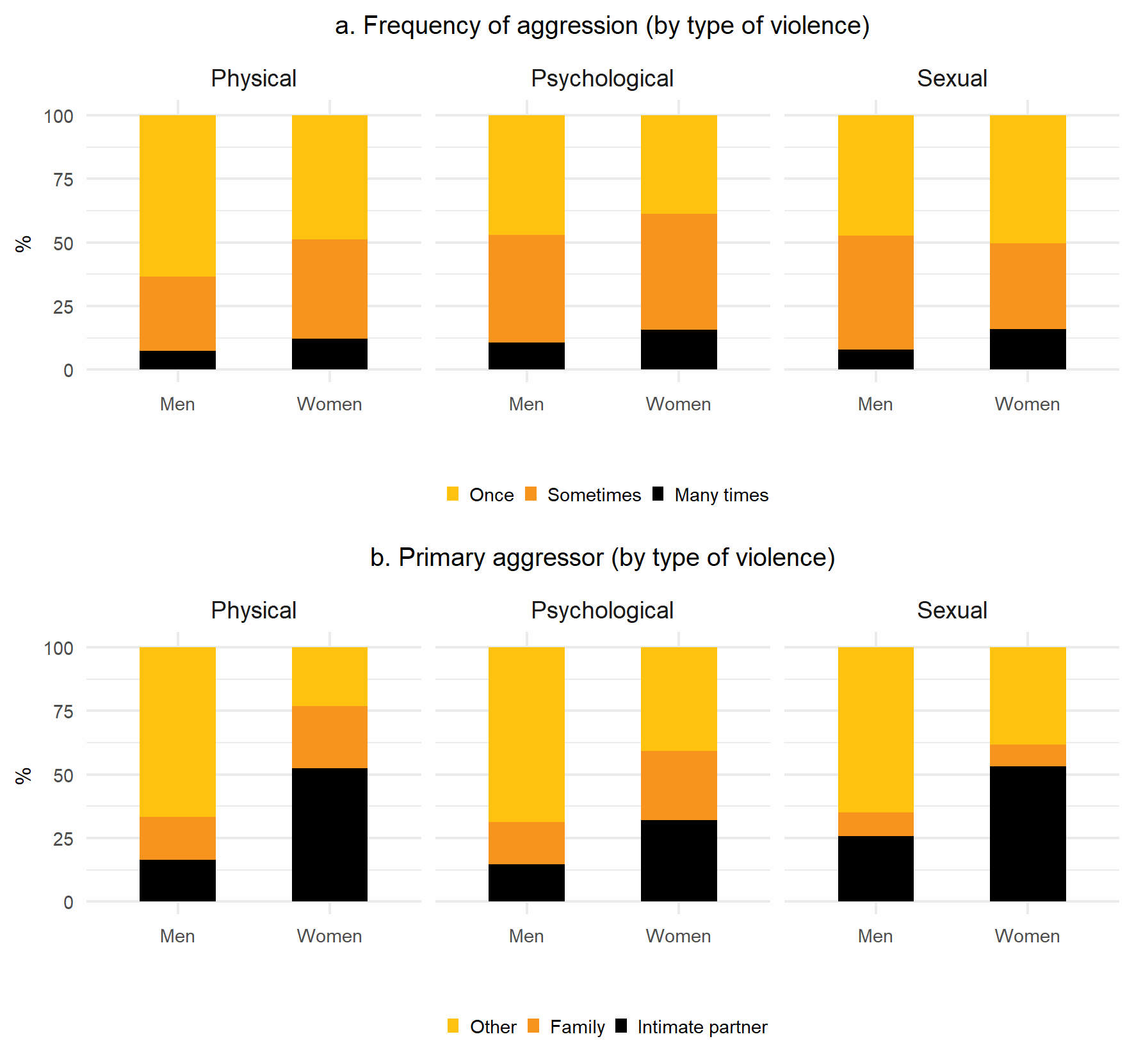


Note: The figure shows, for each type of violence, the frequency that it occurred (panel a) and the primary aggressor (panel b) according to sex/gender. For each type of violence, victimization was defined as being the victim of at least one violent episode in the past 12 months. Intimate partner includes any present or past intimate partner, family includes any family member other than the intimate partner, other known person includes friends/colleagues, employers and/or employees. All data were weighted according to the PNS survey design and survey weights.

### Figure A3 - Inequality patterns in victimization and depression


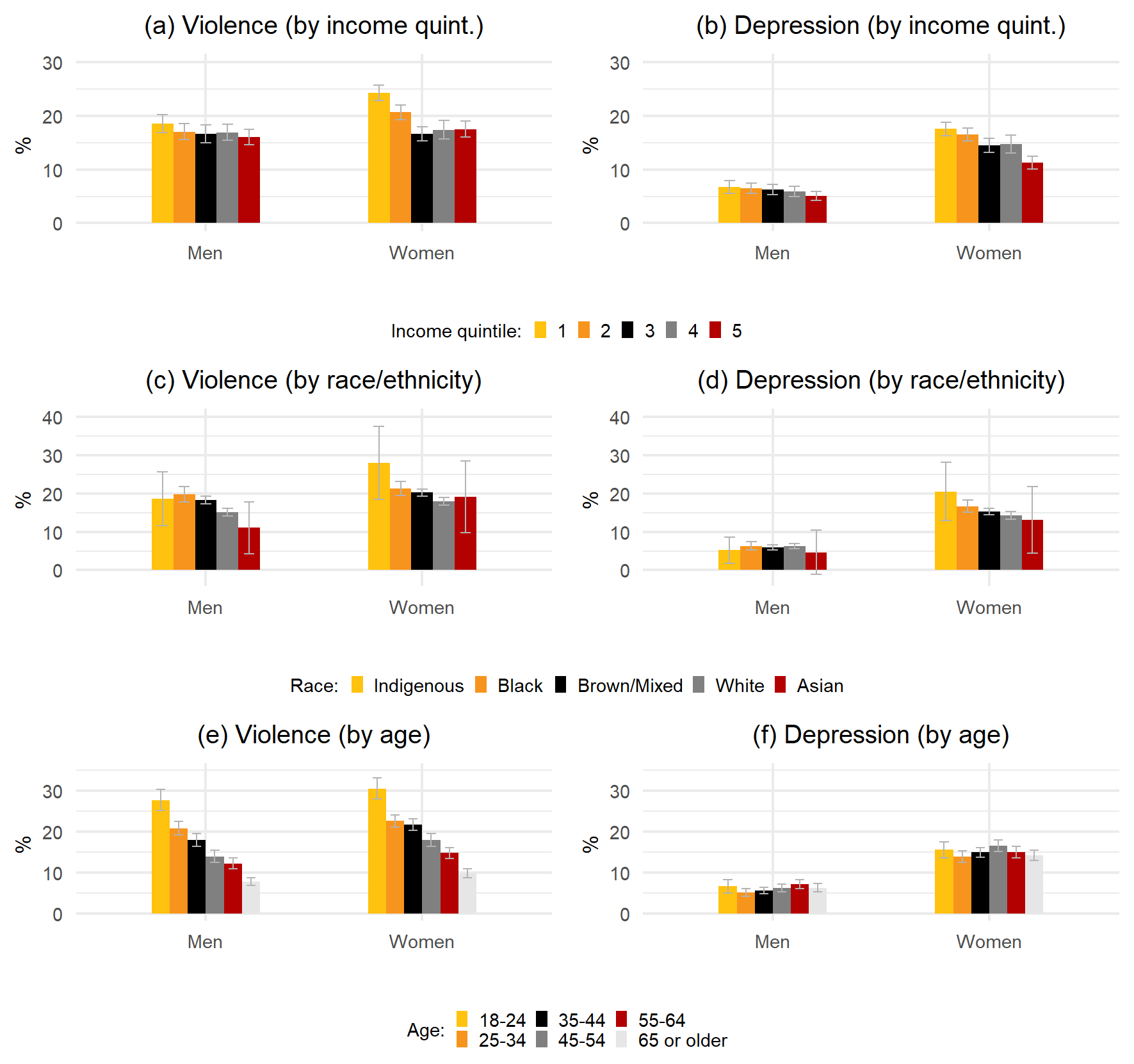


Note: The figure shows the prevalence of victimization and depression by sex/gender and income quintile (panels a and b), by sex/gender and race/ethnicity (panels c and d) and by sex/gender and age (panels e and f). Depression was defined as having a PHQ-9 score >= 10. Victimization was defined as being the victim of at least one violent episode in the past 12 months. Income quintile was defined according to household income per capita. All data were weighted according to the PNS survey design and survey weights.

### Table A2 - Association between observable characteristics and violence victimization

|  | (1) | | (2) | | (3) | | (4) | |
| --- | --- | --- | --- | --- | --- | --- | --- | --- |
|  | Violence (any) | | Psychological violence | | Physical violence | | Sexual violence | |
|  | OR | 95%CI | OR | 95%CI | OR | 95%CI | OR | 95%CI |
|  |  |  |  |  |  |  |  |  |
| Sex (omitted: Men) | 1.331 | 1.238 - 1.430 | 1.351 | 1.254 - 1.455 | 1.300 | 1.123 - 1.505 | 3.215 | 2.336 - 4.426 |
| *Age (omitted: 18-24)* |  |  |  |  |  |  |  |  |
| 25-34 | 0.695 | 0.619 - 0.781 | 0.714 | 0.634 - 0.805 | 0.693 | 0.580 - 0.828 | 0.683 | 0.466 - 1.002 |
| 35-44 | 0.635 | 0.568 - 0.710 | 0.648 | 0.578 - 0.725 | 0.539 | 0.431 - 0.673 | 0.449 | 0.291 - 0.691 |
| 45-54 | 0.496 | 0.438 - 0.562 | 0.519 | 0.456 - 0.589 | 0.397 | 0.318 - 0.496 | 0.665 | 0.341 - 1.297 |
| 55-64 | 0.418 | 0.365 - 0.480 | 0.430 | 0.374 - 0.495 | 0.293 | 0.220 - 0.391 | 0.279 | 0.157 - 0.496 |
| 65 or older | 0.301 | 0.257 - 0.353 | 0.311 | 0.264 - 0.367 | 0.171 | 0.117 - 0.250 | 0.157 | 0.081 - 0.304 |
| *Income quintile (omitted: 1st)* |  |  |  |  |  |  |  |  |
| 2nd | 0.887 | 0.804 - 0.978 | 0.883 | 0.800 - 0.975 | 0.912 | 0.772 - 1.077 | 0.582 | 0.338 - 1.001 |
| 3rd | 0.835 | 0.749 - 0.932 | 0.830 | 0.741 - 0.928 | 0.828 | 0.687 - 0.999 | 0.591 | 0.313 - 1.119 |
| 4th | 0.825 | 0.719 - 0.947 | 0.825 | 0.716 - 0.950 | 0.834 | 0.608 - 1.144 | 0.777 | 0.391 - 1.541 |
| 5th | 0.805 | 0.699 - 0.928 | 0.797 | 0.689 - 0.922 | 0.836 | 0.629 - 1.112 | 0.617 | 0.282 - 1.348 |
| *Race/ethnicity (omitted: White)* |  |  |  |  |  |  |  |  |
| Asian | 0.911 | 0.586 - 1.418 | 0.945 | 0.605 - 1.477 | 0.267 | 0.134 - 0.535 | 0.093 | 0.020 - 0.438 |
| Black | 1.183 | 1.073 - 1.306 | 1.157 | 1.045 - 1.281 | 1.382 | 1.141 - 1.675 | 0.857 | 0.498 - 1.476 |
| Brown/Mixed | 1.131 | 1.046 - 1.224 | 1.125 | 1.037 - 1.220 | 1.338 | 1.156 - 1.548 | 0.760 | 0.491 - 1.177 |
| Indigenous | 1.417 | 0.958 - 2.096 | 1.450 | 0.978 - 2.151 | 1.127 | 0.668 - 1.902 | 0.993 | 0.395 - 2.498 |
| *Region of residence (omitted: South-East)* |  |  |  |  |  |  |  |  |
| Center-West | 0.897 | 0.803 - 1.002 | 0.886 | 0.791 - 0.992 | 0.907 | 0.732 - 1.124 | 1.186 | 0.733 - 1.920 |
| North | 0.903 | 0.806 - 1.012 | 0.880 | 0.783 - 0.989 | 1.010 | 0.830 - 1.229 | 1.106 | 0.654 - 1.869 |
| North-East | 0.991 | 0.902 - 1.088 | 0.978 | 0.890 - 1.076 | 1.004 | 0.845 - 1.193 | 1.131 | 0.669 - 1.910 |
| South | 0.927 | 0.833 - 1.032 | 0.925 | 0.829 - 1.031 | 1.051 | 0.845 - 1.306 | 0.654 | 0.370 - 1.155 |
| *Marital status (omitted: Single)* |  |  |  |  |  |  |  |  |
| Married | 0.872 | 0.793 - 0.959 | 0.876 | 0.793 - 0.966 | 0.792 | 0.654 - 0.959 | 0.493 | 0.233 - 1.041 |
| Divorced | 1.295 | 1.150 - 1.458 | 1.280 | 1.134 - 1.444 | 1.278 | 1.023 - 1.597 | 1.096 | 0.597 - 2.010 |
| Widow | 0.791 | 0.672 - 0.931 | 0.816 | 0.690 - 0.965 | 0.900 | 0.606 - 1.337 | 0.448 | 0.206 - 0.972 |
| *Cohabitation (omitted: Alone)* |  |  |  |  |  |  |  |  |
| Partner | 0.793 | 0.718 - 0.876 | 0.801 | 0.724 - 0.886 | 0.763 | 0.636 - 0.916 | 0.605 | 0.342 - 1.069 |
| Other person | 0.903 | 0.823 - 0.989 | 0.897 | 0.817 - 0.985 | 0.910 | 0.766 - 1.082 | 1.013 | 0.719 - 1.427 |
| *Education (omitted: Basic incomplete)* |  |  |  |  |  |  |  |  |
| Basic | 0.983 | 0.889 - 1.087 | 1.001 | 0.903 - 1.110 | 1.004 | 0.822 - 1.226 | 0.859 | 0.511 - 1.444 |
| Secondary | 0.991 | 0.867 - 1.132 | 1.025 | 0.895 - 1.175 | 0.653 | 0.502 - 0.850 | 0.681 | 0.387 - 1.199 |
| Higher | 0.896 | 0.822 - 0.977 | 0.915 | 0.838 - 1.000 | 0.686 | 0.569 - 0.827 | 0.763 | 0.481 - 1.210 |
| *Physical activity (omitted: No)* |  |  |  |  |  |  |  |  |
| Less than weekly | 1.518 | 1.187 - 1.941 | 1.540 | 1.200 - 1.976 | 1.706 | 1.145 - 2.543 | 0.877 | 0.374 - 2.056 |
| Once or twice a week | 1.142 | 1.037 - 1.259 | 1.133 | 1.026 - 1.252 | 1.104 | 0.904 - 1.348 | 1.346 | 0.901 - 2.013 |
| Three or more times a week | 1.054 | 0.974 - 1.141 | 1.052 | 0.970 - 1.141 | 1.000 | 0.855 - 1.169 | 1.300 | 0.938 - 1.802 |
| *Alcohol consumption (omitted: Never)* |  |  |  |  |  |  |  |  |
| Less than weekly | 1.105 | 1.007 - 1.213 | 1.086 | 0.989 - 1.192 | 1.283 | 1.075 - 1.530 | 1.321 | 0.905 - 1.928 |
| Once a week | 1.115 | 0.999 - 1.245 | 1.112 | 0.994 - 1.243 | 1.132 | 0.934 - 1.371 | 1.096 | 0.704 - 1.704 |
| More than once a week | 1.315 | 1.184 - 1.461 | 1.285 | 1.152 - 1.432 | 1.876 | 1.559 - 2.258 | 2.009 | 1.354 - 2.982 |
|  |  |  |  |  |  |  |  |  |
| Employed (omitted: No) | 1.168 | 1.084 - 1.259 | 1.171 | 1.084 - 1.266 | 0.985 | 0.850 - 1.143 | 1.387 | 0.911 - 2.112 |
| Urban area (omitted: No) | 1.437 | 1.314 - 1.572 | 1.430 | 1.305 - 1.567 | 1.508 | 1.268 - 1.793 | 1.079 | 0.728 - 1.599 |
| Smokes tobacco (omitted: No) | 1.351 | 1.236 - 1.478 | 1.321 | 1.205 - 1.449 | 1.658 | 1.418 - 1.938 | 1.464 | 0.910 - 2.355 |
| Private health insurance (omitted: No) | 0.990 | 0.903 - 1.084 | 0.998 | 0.909 - 1.096 | 0.836 | 0.677 - 1.031 | 0.765 | 0.518 - 1.129 |
| Registered in public primary healthcare (omitted: No) | 0.973 | 0.905 - 1.047 | 0.974 | 0.904 - 1.050 | 0.994 | 0.851 - 1.162 | 1.276 | 0.929 - 1.751 |

Note: The table shows odd ratios (OR) quantifying the association between all covariates included in the analysis and violence victimization (total and by type of violence). Odds ratios were estimated using the results from four different fully adjusted *logit* models. All data were weighted according to the PNS survey design and survey weights.

### Table A3 - Association between observable characteristics and depression

|  | (1) | | (2) | | (3) | | (4) | |
| --- | --- | --- | --- | --- | --- | --- | --- | --- |
|  | Depression | | Depression, by type of violence | | Depression, by primary aggressor | | Depression, by frequency of violence | |
|  | OR | 95%CI | OR | 95%CI | OR | 95%CI | OR | 95%CI |
|  |  |  |  |  |  |  |  |  |
| Violence (omitted: No) | 3.811 | 3.450 - 4.210 |  |  |  |  |  |  |
|  |  |  |  |  |  |  |  |  |
| *Type of violence (omitted: None)* |  |  |  |  |  |  |  |  |
| Only physical |  |  | 1.764 | 1.207 - 2.578 |  |  |  |  |
| Only psychological |  |  | 3.128 | 1.772 - 5.521 |  |  |  |  |
| Only sexual |  |  | 3.494 | 3.155 - 3.870 |  |  |  |  |
| Two types |  |  | 5.591 | 4.521 - 6.913 |  |  |  |  |
| All types |  |  | 8.280 | 5.274 - 12.999 |  |  |  |  |
| *Primary aggressor (omitted: None)* |  |  |  |  |  |  |  |  |
| Family (not partner) |  |  |  |  | 4.458 | 3.817 - 5.207 |  |  |
| Intimate partner violence |  |  |  |  | 4.380 | 3.667 - 5.231 |  |  |
| Other |  |  |  |  | 3.209 | 2.845 - 3.620 |  |  |
| *Frequency of violence (omitted: Never)* |  |  |  |  |  |  |  |  |
| Low |  |  |  |  |  |  | 2.621 | 2.274 - 3.022 |
| Moderate |  |  |  |  |  |  | 3.971 | 3.474 - 4.539 |
| High |  |  |  |  |  |  | 6.100 | 5.188 - 7.172 |
|  |  |  |  |  |  |  |  |  |
| Sex (omitted: Men) | 2.340 | 2.116 - 2.588 | 2.316 | 2.094 - 2.560 | 2.267 | 2.050 - 2.506 | 2.303 | 2.084 - 2.545 |
| *Age (omitted: 18-24)* |  |  |  |  |  |  |  |  |
| 25-34 | 1.026 | 0.866 - 1.215 | 1.028 | 0.866 - 1.219 | 1.019 | 0.860 - 1.207 | 1.020 | 0.860 - 1.209 |
| 35-44 | 1.118 | 0.943 - 1.325 | 1.136 | 0.958 - 1.348 | 1.116 | 0.941 - 1.323 | 1.120 | 0.945 - 1.327 |
| 45-54 | 1.256 | 1.055 - 1.495 | 1.268 | 1.063 - 1.513 | 1.252 | 1.051 - 1.492 | 1.247 | 1.047 - 1.486 |
| 55-64 | 1.083 | 0.893 - 1.314 | 1.105 | 0.911 - 1.340 | 1.084 | 0.894 - 1.315 | 1.083 | 0.892 - 1.314 |
| 65 or older | 0.911 | 0.740 - 1.121 | 0.928 | 0.754 - 1.142 | 0.907 | 0.737 - 1.117 | 0.912 | 0.740 - 1.123 |
| *Income quintile (omitted: 1st)* |  |  |  |  |  |  |  |  |
| 2nd | 1.020 | 0.904 - 1.151 | 1.027 | 0.909 - 1.161 | 1.024 | 0.907 - 1.156 | 1.032 | 0.914 - 1.166 |
| 3rd | 0.937 | 0.821 - 1.069 | 0.946 | 0.829 - 1.080 | 0.944 | 0.827 - 1.077 | 0.949 | 0.831 - 1.084 |
| 4th | 0.993 | 0.849 - 1.161 | 0.998 | 0.855 - 1.164 | 0.997 | 0.852 - 1.166 | 1.004 | 0.860 - 1.173 |
| 5th | 0.840 | 0.702 - 1.006 | 0.846 | 0.707 - 1.011 | 0.843 | 0.705 - 1.008 | 0.854 | 0.714 - 1.022 |
| *Race/ethnicity (omitted: White)* |  |  |  |  |  |  |  |  |
| Asian | 0.793 | 0.435 - 1.444 | 0.810 | 0.444 - 1.477 | 0.786 | 0.437 - 1.414 | 0.807 | 0.433 - 1.506 |
| Black | 1.020 | 0.896 - 1.160 | 1.023 | 0.899 - 1.164 | 1.025 | 0.900 - 1.166 | 1.021 | 0.897 - 1.162 |
| Brown/Mixed | 0.959 | 0.875 - 1.052 | 0.956 | 0.871 - 1.049 | 0.961 | 0.876 - 1.054 | 0.954 | 0.869 - 1.048 |
| Indigenous | 1.077 | 0.693 - 1.675 | 1.083 | 0.699 - 1.678 | 1.068 | 0.681 - 1.675 | 1.078 | 0.690 - 1.686 |
| *Region of residence (omitted: South-East)* |  |  |  |  |  |  |  |  |
| Center-West | 1.016 | 0.895 - 1.154 | 1.019 | 0.898 - 1.156 | 1.015 | 0.894 - 1.153 | 1.023 | 0.901 - 1.160 |
| North | 0.702 | 0.616 - 0.800 | 0.703 | 0.616 - 0.801 | 0.700 | 0.615 - 0.798 | 0.706 | 0.619 - 0.805 |
| North-East | 0.896 | 0.807 - 0.996 | 0.899 | 0.808 - 1.000 | 0.897 | 0.807 - 0.997 | 0.901 | 0.810 - 1.002 |
| South | 0.923 | 0.808 - 1.054 | 0.923 | 0.809 - 1.054 | 0.923 | 0.809 - 1.054 | 0.923 | 0.808 - 1.055 |
| *Marital status (omitted: Single)* |  |  |  |  |  |  |  |  |
| Married | 1.012 | 0.906 - 1.130 | 1.021 | 0.913 - 1.141 | 1.015 | 0.909 - 1.134 | 1.017 | 0.911 - 1.136 |
| Divorced | 1.197 | 1.040 - 1.378 | 1.205 | 1.046 - 1.387 | 1.191 | 1.034 - 1.371 | 1.197 | 1.039 - 1.379 |
| Widow | 0.961 | 0.832 - 1.110 | 0.963 | 0.834 - 1.112 | 0.960 | 0.831 - 1.109 | 0.964 | 0.833 - 1.115 |
| *Cohabitation (omitted: Alone)* |  |  |  |  |  |  |  |  |
| Partner | 0.877 | 0.767 - 1.002 | 0.878 | 0.767 - 1.004 | 0.868 | 0.759 - 0.992 | 0.878 | 0.767 - 1.004 |
| Other person | 1.030 | 0.916 - 1.157 | 1.029 | 0.916 - 1.156 | 1.023 | 0.911 - 1.149 | 1.031 | 0.917 - 1.159 |
| *Education (omitted: Basic incomplete)* |  |  |  |  |  |  |  |  |
| Basic | 0.916 | 0.800 - 1.049 | 0.906 | 0.791 - 1.039 | 0.912 | 0.797 - 1.045 | 0.915 | 0.799 - 1.049 |
| Secondary | 0.836 | 0.713 - 0.981 | 0.841 | 0.717 - 0.987 | 0.844 | 0.720 - 0.990 | 0.853 | 0.727 - 1.001 |
| Higher | 0.810 | 0.723 - 0.908 | 0.814 | 0.727 - 0.912 | 0.815 | 0.727 - 0.912 | 0.822 | 0.734 - 0.920 |
| *Physical activity (omitted: No)* |  |  |  |  |  |  |  |  |
| Less than weekly | 1.077 | 0.767 - 1.514 | 1.062 | 0.752 - 1.498 | 1.062 | 0.757 - 1.491 | 1.080 | 0.760 - 1.534 |
| Once or twice a week | 0.659 | 0.569 - 0.763 | 0.656 | 0.567 - 0.761 | 0.659 | 0.570 - 0.763 | 0.660 | 0.570 - 0.764 |
| Three or more times a week | 0.674 | 0.605 - 0.751 | 0.674 | 0.605 - 0.750 | 0.674 | 0.604 - 0.751 | 0.675 | 0.606 - 0.752 |
| *Alcohol consumption (omitted: Never)* |  |  |  |  |  |  |  |  |
| Less than weekly | 0.900 | 0.800 - 1.013 | 0.899 | 0.798 - 1.011 | 0.897 | 0.796 - 1.010 | 0.903 | 0.802 - 1.017 |
| Once a week | 0.811 | 0.703 - 0.936 | 0.811 | 0.702 - 0.936 | 0.812 | 0.704 - 0.936 | 0.814 | 0.705 - 0.940 |
| More than once a week | 0.835 | 0.725 - 0.962 | 0.818 | 0.708 - 0.944 | 0.831 | 0.721 - 0.957 | 0.830 | 0.720 - 0.957 |
|  |  |  |  |  |  |  |  |  |
| Employed (omitted: No) | 0.657 | 0.600 - 0.718 | 0.657 | 0.600 - 0.718 | 0.660 | 0.603 - 0.722 | 0.659 | 0.602 - 0.721 |
| Urban area (omitted: No) | 1.540 | 1.375 - 1.725 | 1.542 | 1.377 - 1.728 | 1.548 | 1.383 - 1.734 | 1.538 | 1.373 - 1.723 |
| Smokes tobacco (omitted: No) | 1.507 | 1.344 - 1.691 | 1.500 | 1.335 - 1.685 | 1.500 | 1.337 - 1.683 | 1.485 | 1.322 - 1.668 |
| Private health insurance (omitted: No) | 1.055 | 0.934 - 1.192 | 1.062 | 0.943 - 1.196 | 1.062 | 0.941 - 1.198 | 1.058 | 0.937 - 1.194 |
| Registered in public primary healthcare (omitted: No) | 1.059 | 0.965 - 1.162 | 1.055 | 0.962 - 1.158 | 1.058 | 0.964 - 1.161 | 1.053 | 0.960 - 1.155 |

Note: The table shows odd ratios (OR) quantifying the association between all covariates included in the analysis and depression. Odds ratios were estimated using the results from four different fully adjusted logit models. In each model, a different variable was used to measure violence victimization (i.e., total, by type of violence, by primary aggressor, and by frequency of violence). All data were weighted according to the PNS survey design and survey weights.

### Figure A4- Predicted probabilities for falling in the treatment gap for depression according to sex/gender and victimization


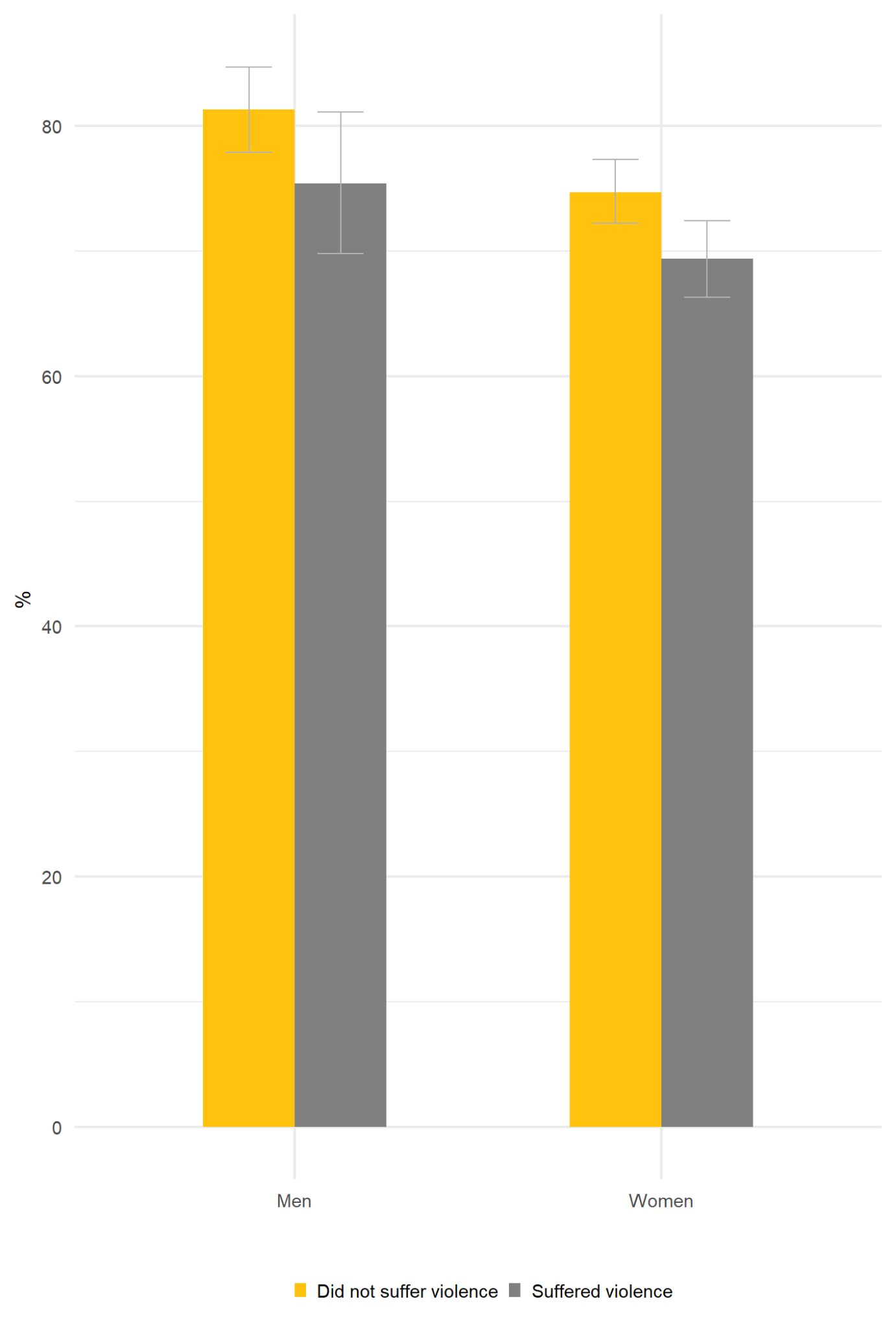


Note: The figure shows the predicted probabilities for falling in the treatment gap (i.e., being untreated, conditional on having depression) according to sex/gender, and violence victimization. Victimization was defined as being the victim of at least one violent episode in the past 12 months. Falling in the treatment gap was defined as having a PHQ-9 score >= 10 and not receiving any medical treatment for depression. Predicted probabilities were estimated using the results of logit models that included as covariates: sex/gender, victimization, the interaction term between sex/gender and victimization, income quintile, race/ethnicity, age, region of residence, area of residence (urban/rural), marital status, cohabitation, employment, education, alcohol consumption, tobacco consumption, physical activity, health insurance and registered in primary healthcare services. All other covariates were fixed at means. All data were weighted according to the PNS survey design and survey weights.
